# Laparoscopic resection for a case preoperatively diagnosed as adrenal rest tumor in the liver: A case report

**DOI:** 10.1016/j.ijscr.2024.110408

**Published:** 2024-10-02

**Authors:** Masataka Nakagawa, Tomoyuki Abe, Tsuyoshi Kobayashi, Hideki Ohdan, Kazuhiro Toyota

**Affiliations:** a Department of Gastroenterological Surgery, National Hospital Organization Higashihiroshima Medical Center, 513 Jike, Saijocho, Higashihiroshima 739-0041, Japan; b Department of Gastroenterological Surgery, Hiroshima University Hospital, 1-2-3 Kasumi, Minami-ku, Hiroshima 734-8551, Japan

**Keywords:** Adrenal rest tumor, Hepatocellular carcinoma, Laparoscopic resection

## Abstract

**Introduction and importance:**

Adrenal rest tumors (ARTs) are frequently found in the abdominal axis and testis and are often detected incidentally during surgery or autopsy. The standard treatment is complete resection due to their malignant potential; however, precise preoperative diagnosis is difficult due to the similarity of the radiological findings of this disease with those of hepatocellular carcinoma (HCC) and angiomyolipoma.

**Case presentation:**

A 58-year-old woman was diagnosed with a tumor through a physical examination and came to our clinic for a close examination. Dynamic computed tomography showed a tumor with a diameter of 27 mm that occupied segment 7 (S7) of the liver. The tumor was slightly enhanced in the arterial phase and washed out in the portal phase. Magnetic resonance imaging (MRI) demonstrated that this round tumor protruded from the liver surface and had high signal intensity on T2-weighted imaging. The tumor showed a high signal intensity on diffusion-weighted imaging. Chemical shift imaging revealed the markedly low intensity of the tumor. The preoperative diagnosis was suspected hepatic ART. Laparoscopic S7 partial resection was performed. The operative duration was 147 min, and the blood loss was 10 mL. The patient was discharged from the hospital on postoperative day 5. The pathological diagnosis was ART.

**Clinical discussion:**

Hepatic ART (HART) is diagnosed preoperatively as HCC in most cases, and resection is rarely reported. The most standard treatment for HART is surgery for possible malignancy. Recent advances in radiological imaging have made it possible to distinguish HART from HCC using MRI chemical shift images. In the case of a highly vascular and fatty tumor such as the present case, HART diagnosis can be made preoperatively using MRI chemical shift imaging. This case is the first reported preoperative diagnosis of HART using chemical shift imaging.

**Conclusions:**

We report a case of laparoscopic radical resection in a patient preoperatively diagnosed with hepatic ART. Chemical shift imaging in MRI is essential for distinguishing ART from HCC.

## Highlights

• Preoperative diagnosis of adrenal rest tumors is difficult.
• We preoperatively diagnosed an adrenal rest tumor using chemical shift imaging.
• Chemical shift imaging in MRI can distinguish adrenal rest tumor from other cancers.

## 1 Introduction

Adrenal rest tumors (ARTs) are rare tumors that occur in various organs, including the liver, testes, and ovaries [1]. Adrenal rests usually consist of only adrenal cortical cells, rarely containing adrenal medullary cells. Aberrant adrenal tissues are divided into the heterotopic and accessory adrenal glands. Heterotropic adrenal glands are derived from the adrenal primordium that has migrated at an embryonic stage from neighboring organs such as the kidneys or the liver, whereas accessory adrenal glands are the ectopia of fragmented adrenal tissues into the celiac axis, retroperitoneal cavity, uterus, broad ligament, or testes. Rarely, ARTs occur in the liver. Hepatic ARTs (HARTs) appear as fat-containing hypervascular masses on imaging. HARTs are difficult to distinguish from hepatocellular carcinoma (HCC) and angiomyolipoma owing to their radiological similarities [2]. Most cases of HART are incidentally detected during surgery or autopsy [1]. The incidence of HART is low, but this disease has malignant potential, and complete resection is the gold-standard treatment. Common symptoms of ART include high fever and hypertension, which are caused by hormone production. Herein, we report a case of laparoscopic S7 partial resection for a patient preoperatively diagnosed with HART. This case is reported according to the SCARE 2023 criteria [3].

## 2 Presentation of case

**Fig. 1:**
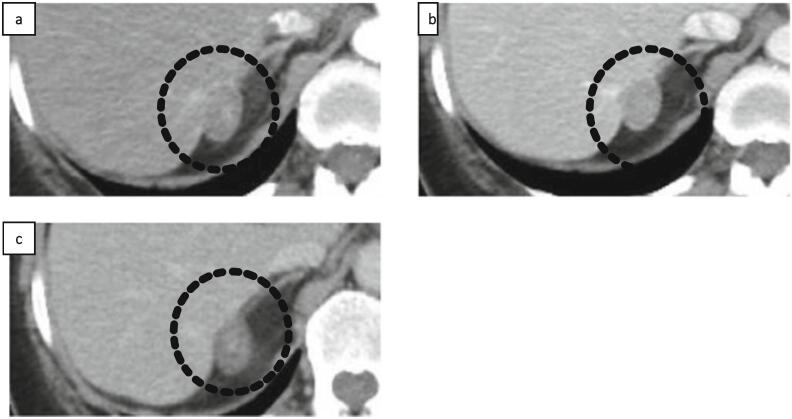
(a) Enhanced computed tomography (CT) revealed a 27-mm well-defined tumor located at liver segment 7, which was enhanced in the arterial phase. The enhancement was washed out in the (b) portal and (c) delayed phase (indicated by black dotted circle).

**Fig. 2:**
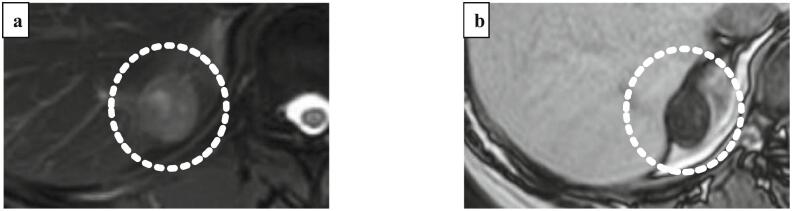
(a) On magnetic resonance imaging (MRI), T2-weighted fat-suppressed imaging showed that there was high intensity at the tumor, (b) chemical shift imaging showed markedly low intensity at the tumor (indicated by white dotted circle).

A 58-year-old woman was referred to our hospital due to the presence of a hepatic mass after an annual physical examination. A primary malignant liver tumor was suspected, and further examination was performed. The patient had no relevant medical history. Her family history was unremarkable. Laboratory investigations showed no elevation in serum alpha-fetoprotein or protein levels induced by vitamin K absence/antagonist II. The hepatitis B surface antigen and hepatitis C antibody test results were negative. The aspartate aminotransferase level was 23 IU/L, alanine aminotransferase level was 27 IU/L, alkaline phosphatase level was 80 IU/L, and γ-glutamyl transpeptidase level was 79 IU/L. The total bilirubin level was 0.7 mg/dL and the albumin level was 4.4 g/dL. Enhanced computed tomography (CT) revealed a 27-mm well-defined tumor located at segment 7 (S7) of the liver, which was enhanced in the arterial phase (Fig. 1a) and the enhancement was washed out in the portal (Fig. 1b) and delayed phases (Fig. 1c). On magnetic resonance imaging (MRI), T2-weighted fat-suppressed imaging showed that there was high intensity at the tumor (Fig. 2a). Chemical shift imaging revealed the tumor exhibited markedly low intensity (Fig. 2b).

**Fig. 3:**
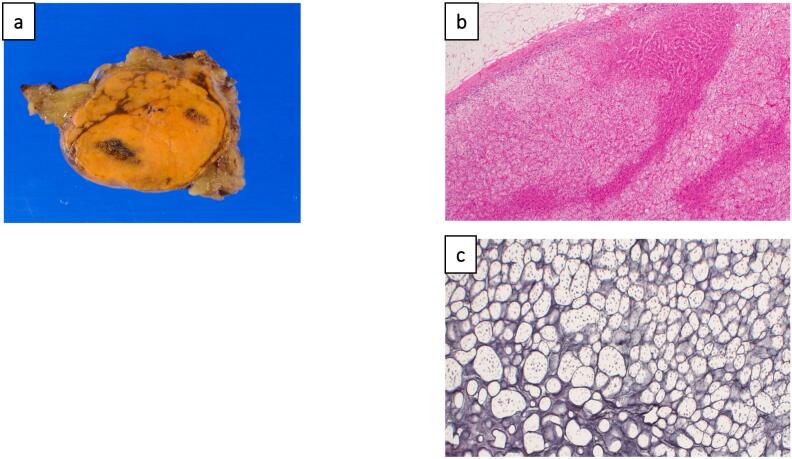
Pathological findings of the resected tumor. (a) Pathological examination revealed the tumor was surrounded by a thin fibrous capsule on macroscopic findings. (b) Hematoxylin–eosin (HE) staining showed that the lesion was composed of cells with a micro-vesicular cytoplasm, and these were aligned in alveolar or trabecular arrangements. (c) Foci formation was similar to that of adrenocortical tissue on silver plating.

Based on the radiological findings, HART was suspected. Laparoscopic S7 partial hepatectomy was performed. Intraoperatively, the tumor was clearly separated to the normal right adrenal gland, and the right adrenal gland was preserved. The operative duration was 147 min and the blood loss was 10 mL. The patient was discharged from the hospital on postoperative day 5 without any complications. The tumor was surrounded by a thin fibrous capsule on macroscopic findings (Fig. 3a). The lesion was composed of cells with a microvascular cytoplasm, and these were aligned in alveolar or trabecular arrangements (Fig. 3b). Foci formation was similar to that of adrenocortical tissue on silver plating (Fig. 3c). Malignant features were not detected in the adrenal cortex. The Ki-67 proliferation index of the tumor cells was 1–2 %. Pathological examination led to the diagnosis of HART.

## 3 Discussion

HART has radiological findings similar to those of HCC and angiomyolipoma, making accurate preoperative diagnosis difficult [2]. This is because the hepatic artery directly supplies blood to the tumor; therefore, the early enhancement at the early phase and the wash-out of this enhancement in the late phase quietly mimic the presentation of HCC. It also reflects the presence of fat droplets in the cytoplasm of tumor cells [2,5]. Angiomyolipomas are similar, with no definitive imaging features that clearly distinguish them from HART. They typically present as round or oval masses with well-defined borders, low density, and minimal contrast enhancement. In previous reports, most cases of HART were preoperatively diagnosed as HCC. Table 1 shows 12 resection cases for HART [1,2,[4], [5], [6], [7], [8], [9], [10], [11], [12], [13]]. Regarding the surgical approach, ours is the only case in which the tumor was resected laparoscopically. Several contributed to the decision to perform laparoscopic liver resection in this case. First, the patient had no history of prior abdominal surgery, and the tumor's prominent protrusion from the liver surface made it suitable for laparoscopic surgery. Laparoscopic liver resection has demonstrated significant advantages over open surgery, including shorter hospital stays and reduced postoperative pain. Furthermore, the absence of hormone overproduction in this case further supported the choice of a laparoscopic approach. HART is more common in younger patients, with no sex differences [2]. It is most commonly located in S7 of the right lobe of the liver [1,2,[4], [5], [6], [7], [8], [9], [10], [11], [12], [13]]. Ectopic adrenal tissue is often a benign adenoma; however, rare malignant cases have been reported. Functional tumors can cause masculinization or Cushing's syndrome, and immunohistochemical detection of cortisol and testosterone in tumor cells has been reported [4,5]. Two cases have been reported with malignant histological features [6,7]. Ten cases were reported as nonfunctional and presented with incidental findings [1,2,4,5,[8], [9], [10], [11], [12], [13]].

**Table 1:** Summary of previous reported cases with ART following complete resection.

Authors and year of publication	Age (years)	Sex	Size (mm)	Location	Functional status	Malignant transformation
Hyams et al. [5]	59	M	30	Right liver	Non-functional	No
Wallace et al. [6]	23	F	150	Right liver	Functional	Yes
Contreras et al. [7]	21	F	100	S7	Functional	Yes
Arai et al. [8]	62	M	25	S7	Non-functional	No
Tajima et al. [2]	55	F	25	S7	Non-functional	No
Baba et al. [4]	67	F	15	S7	Non-functional	No
Shin et al. [9]	62	M	30	S7	Non-functional	No
Dalla Valle et al. [10]	58	M	25	S7	Non-functional	No
Soo et al. [11]	47	F	34	S7	Non-functional	No
Sugiyama et al. [12]	50	F	30	S7	Non-functional	No
Enjoji et al. [1]	67	F	17	S7	Non-functional	No
Al-Taee et al. [13]	72	F	23	S7	Non-functional	No
our cases	58	F	21	S7	Non-functional	No

Recent advancements in radiological imaging have made it possible to distinguish between HART and HCC using chemical shift imaging in MRI. Typical MRI features of early HCC are iso- to hyperintensity on T1-weighted images and hypointensity on T2-weighted images [14]. Early-stage HCC appears hypointense in the hepatobiliary phase on gadolinium ethoxybenzyl diethylenetriamine pentaacetic acid-enhanced MRI [15]. However, the preoperative diagnosis of HART was confirmed by a pale heterogeneous high signal on fat-suppressed T2-weighted images and a prominent low signal on chemical shift imaging. Some reports suggest that chemical shift imaging in MRI is the key to differentiating HCC from HART [16,17]. In MRI chemical shift imaging, the signal intensities generated from water and lipid protons are additive on in-phase chemical shift images and subtractive on opposed-phase chemical shift images [15].

In this case, as in other reported cases, the tumor exhibited hyperintensity on T2-weighted fat-suppressed images but was markedly hypointense on chemical shift images, leading to the diagnosis of HART. Savci et al. found that the use of subtraction techniques with chemical shift MRI significantly enhanced the visualization of adrenal masses, improving both quantitative and qualitative assessments [16]. This approach allows for better diagnostic accuracy, especially in distinguishing between benign and malignant lesions.

However, it is not clear whether MRI is necessary in addition to CT [16,17]. There are several reasons for this. The quantitative analysis of chemical shift images requires calculations based on ratios and other formulas, which can be cumbersome in routine radiology practice. When visual analysis of inverse and in-phase images is performed, the reduction in signal intensity in tumors is not always obvious and requires considerable reading experience [16]. However, fatty HCC is often highly differentiated and presents with poor hepatic arterial perfusion; therefore, in the case of hypervascular and fatty tumors, such as in the present case, preoperative HART diagnosis could be possible using chemical shift imaging in MRI. Our case is the first to report a preoperative diagnosis of HART using chemical shift imaging [1,2,[4], [5], [6], [7], [8], [9], [10], [11], [12], [13]]. Ectopic adrenal tissue can be embryologically heterotopic or an accessory adrenal gland. Heterotopia is the result of the early developmental migration of the adrenal gland into adjacent organs, such as the liver and kidneys. An accessory adrenal gland is the late embryonic stage of adrenal gland migration, when fragmented adrenal tissue is transferred. In each case, only the adrenal cortex is involved without adrenal medullary tissue of different embryological origin [4]. ART is most commonly detected in the testes, ovaries, and broad ligaments, but its frequency is low [18]. Testicular ART (TART) is common in men with congenital adrenal hyperplasia, whereas ovarian ART (OART) is rare. TART and OART are not malignant tumors, but can cause the dysfunction of nearby tissues. TART can cause irreversible damage to the surrounding testicular tissue and may result in gonadal dysfunction and infertility [19]. Disruption of the hypothalamic–pituitary–ovarian axis due to excessive androgen and progesterone secretion in patients with OART can causes infertility [18].

The gold standard treatment for HART is surgery to account for the malignant potential of the disease. Surgery is also considered for TART and OART, which can cause gonadal dysfunction. Nakamura et al. stated that if histopathology shows no evidence of malignancy, the patient should be followed up with using blood and imaging tests every 3 months [20].

## 4 Conclusion

We encountered a case in which HART was diagnosed preoperatively and removed laparoscopically.

## Consent

Written informed consent was obtained from the patient for publication and any accompanying images. A copy of the written consent is available for review by the Editor-in-Chief of this journal on request.

## Ethical approval

Ethics approval for this study was granted by the Ethics Committee NAC of East Hiroshima Medical Center on 07/29/2024.

## Funding

No funding.

## Author contribution

MN drafted the manuscript. TA supervised drafting of the manuscript.TK, HO and KT performed the surgical procedure and perioperative management. All the authors have read and approved the final version of the manuscript.

## Guarantor

Masataka Nakagawa, Tomoyuki Abe, Tsuyoshi Kobayashi, Hideki Ohdan, Kazuhiro Toyota.

## Conflict of interest statement

The authors declare that they have no competing interest.
